# Perceptions of Equity, Loneliness, and Depressive Symptoms among Siblings Who Care for an Older Parent with Dementia

**DOI:** 10.1002/alz70858_097636

**Published:** 2025-12-24

**Authors:** Megan Gilligan, Hanamori F Skoblow, Jeenkyoung F Lee, Destiny Ogle

**Affiliations:** ^1^ University of Missouri, Columbia, MO, USA; ^2^ Purdue University, West Lafayette, IN, USA

## Abstract

**Background:**

Over half of caregivers for individuals living with Alzheimer's disease and related dementias (ADRD) are adult children. Extensive research indicates that adult child caregivers are at an elevated risk for several health problems, and that such issues are particularly severe among caregivers of parents living with ADRD. This research highlights the role of family relations in caregivers’ well‐being by considering the sibling network in which adult child caregivers are often embedded. Equity theory proposes that individuals are the most satisfied with relationships in which they experience relatively equal exchanges rather than being over benefited or under benefited. Equity theorists argue that individuals tend to feel anger and resentment when under benefited, and guilt when over benefited. Research indicates that perceived imbalance in interpersonal relationships negatively impacts mental health outcomes; however, the association between perceptions of equity in care provision among siblings and caregivers’ mental health has received little attention. Thus, we extend the literature by examining the association between perceptions of care equity, loneliness, and depressive symptoms among siblings in which an older parent is living with ADRD.

**Method:**

Data came from 208 adult child caregivers (*M*_age_ = 58) nested in 111 families in which an older parent is living with ADRD. Adult children reported their perceptions of care equity using three response categories (1 = *your sibling helps more*, 2 = *you and your sibling help equally*, 3 = *you help more*). Caregiver mental health was measured using the UCLA Loneliness scale and the Center for Epidemiologic Studies Depression scale.

**Result:**

Regression analyses with cluster robust standard errors showed that individuals who perceived themselves as providing more care than their sibling reported higher levels of loneliness compared to those who perceived that care was equitably distributed. Caregivers reported greater depressive symptoms both when they perceived providing more and less care than their siblings, relative to those who reported equitable care.

**Conclusion:**

Our findings highlight the importance of equity in the division of caregiving responsibilities to promote better mental well‐being among caregivers.

